# Extracellular Vesicles as Mediators of Endothelial and Tubular Injury in Cardiac Surgery-Associated Acute Kidney Injury

**DOI:** 10.3390/biomedicines14050982

**Published:** 2026-04-24

**Authors:** Elena Grossini, Teresa Esposito, Mohammad Mostafa Ola Pour, Carlo Smirne, Giovanni Casali, Mario Pirisi, Vincenzo Cantaluppi, Daniele Pierelli, Rosanna Vaschetto, Sakthipriyan Venkatesan

**Affiliations:** 1 Laboratory of Physiology, Department of Translational Medicine, Università Del Piemonte Orientale, Via Solaroli 17, 28100 Novara, Italy; elena.grossini@med.uniupo.it (E.G.);; 2 Division of Cardiac Anesthesiology, AOU Maggiore Della Carità Hospital, Corso Mazzini 18, 28100 Novara, Italy; 3 Internal Medicine Unit, Department of Translational Medicine, Università Del Piemonte Orientale, Via Solaroli 17, 28100 Novara, Italy; 4 Cardiac Surgery, AOU Maggiore Della Carità Hospital, Corso Mazzini 18, 28100 Novara, Italy; 5 Nephrology and Kidney Transplantation Unit, Department of Translational Medicine, AOU Maggiore Della Carità Hospitail, Università Del Piemonte Orientale, 28100 Novara, Italy; 6 Anaesthesiology Unit, Department of Translational Medicine, Università Del Piemonte Orientale, Via Solaroli 17, 28100 Novara, Italy; rosanna.vaschetto@med.uniupo.it

**Keywords:** cardiac failure, endothelial dysfunction, extracellular vesicles, mitochondria, oxidative stress, tubular injury

## Abstract

Cardiac surgery represents a cornerstone of modern cardiovascular medicine, yet it is intrinsically linked to significant systemic stress responses that can compromise remote organ function. Among postoperative complications, cardiac surgery-associated acute kidney injury (CSA-AKI) remains a significant clinical challenge characterized by high morbidity and complex pathophysiology. While hemodynamic instability and ischemia–reperfusion injury are established risk factors, renal dysfunction frequently persists despite optimal perfusion. This observation suggests the involvement of potent circulating mediators in cellular injury. Extracellular vesicles (EVs) are essential for intercellular communication and serve as central hubs for transporting bioactive lipids, proteins, and genetic material. Accumulating evidence indicates that the mechanical and oxidative stress inherent to cardiopulmonary bypass triggers substantial release of EVs from platelets, erythrocytes, and injured vascular tissues. These vesicles may function as vectors that traffic oxidized mitochondrial components and pro-inflammatory cargo to the renal parenchyma. This signaling cascade appears to disrupt renal homeostasis through a proposed “dual-hit” mechanism involving the induction of endothelial dysfunction and endothelial-to-mesenchymal transition (EndMT), followed by tubular epithelial injury via mitochondrial fragmentation, redox imbalance, and downregulation of anti-aging factors. The complexity of these EV-mediated interactions may contribute to an incomplete understanding of why specific patient phenotypes fail to recover. This narrative review examines the mechanisms of surgery-induced EV biogenesis, the molecular pathogenesis of endothelial and tubular damage, and the role of intercellular crosstalk. Additionally, we discuss future perspectives on targeting the “EV vector” through therapeutic apheresis and mitochondrial pharmacotherapy to potentially improve clinical outcomes in high-risk surgical patients.

## 1. Introduction

Cardiac surgery utilizing cardiopulmonary bypass (CPB) remains a cornerstone of modern cardiovascular medicine. However, despite advancements in surgical techniques and perioperative care, the systemic stress response induced by CPB continues to compromise remote organ function. Among postoperative complications, cardiac surgery-associated acute kidney injury (CSA-AKI) represents a significant clinical challenge, affecting up to 30% of patients and necessitating renal replacement therapy in 2% to 5% of cases [1]. CSA-AKI is not merely a transient complication; it is an independent predictor of morbidity that extends intensive care unit stays and increases healthcare costs. Critically, it elevates the risk of short-term mortality by nearly five-fold and predisposes survivors to long-term chronic kidney disease (CKD) [2]. The lack of effective pharmacological treatments highlights a critical gap in our understanding of the underlying molecular pathophysiology.

Historically, the pathophysiology of CSA-AKI has been attributed primarily to hemodynamic perturbations. The kidney operates near the threshold of dysoxia even under physiological conditions due to the high metabolic demand of tubular reabsorption [3]. Therefore, renal injury was logically ascribed to hypotension, non-pulsatile flow, and renal hypoperfusion during the CPB [4]. The influence of perfusion pressure and pump flow during CPB on renal outcomes remains equivocal. Prospective studies demonstrate that increasing CPB flow and mean arterial pressure improves renal medullary oxygenation [5,6], yet targeting higher arterial pressure during CPB has not translated into a consistent reduction in CSA-AKI in meta-analyses of randomized trials, where renal endpoints were frequently secondary [7]. This discrepancy indicates that hemodynamic optimization, while necessary, is insufficient on its own to prevent CSA-AKI, pointing to the concurrent involvement of non-hemodynamic, circulating mediators generated by the extracorporeal circuit [8].

To identify these non-hemodynamic mediators, one must examine the bio-incompatibility of the extracorporeal circuit itself. CPB requires routing the patient’s blood through an artificial extracorporeal circuit to oxygenate and pump it, bypassing the heart and lungs. The contact of blood with these artificial surfaces triggers the contact activation system, a cascade of plasma proteins that initiates immediate coagulation and inflammation, including the complement and kallikrein–kinin pathways [9]. Concurrently, pump rollers and suction tips impose non-physiological shear stress on blood cells. This trauma, combined with hyperoxia, creates a milieu of profound oxidative stress [10]. In this environment, the erythrocyte becomes an important source of toxicity. Shear stress induces hemolysis, releasing free hemoglobin and catalytic iron into the plasma [11]. While scavenger proteins normally sequester these toxins, evidence suggests these defenses can be saturated during CPB. The accumulation of cell-free hemoglobin scavenges nitric oxide (NO), potentially leading to vasoconstriction, and may precipitate in renal tubules, driving oxidative injury via the Fenton reaction [12]. However, free hemoglobin and soluble cytokines alone do not fully account for the specific, sustained signal transduction observed in injured renal cells. Soluble molecules are often rapidly diluted or degraded in the bloodstream. To explain the persistence of injury and the metabolic reprogramming seen in the kidney, a stable vehicle capable of protecting these molecules and delivering them intracellularly would be required.

In this context, extracellular vesicles (EVs), which are lipid bilayer-enclosed nanoparticles typically 30 to 1000 nm in diameter, have emerged as the primary candidates. Once dismissed as cellular debris, EVs are now recognized as essential mediators of intercellular communication [13]. Encapsulated within a lipid bilayer, EVs carry cargo including bioactive lipids, proteins, and genetic material [(e.g., mitochondrial deoxyribonucleic acid (mtDNA), micro ribonucleic acid (microRNA)] derived from their parent cells [14]. This membrane protects the cargo from degradation, allowing EVs to function as stable vectors that travel systemically and alter the phenotype of recipient cells [15]. This concept of EV-mediated systemic pathology is not isolated to CSA-AKI; it mirrors established mechanisms in tumor metastasis, where EVs prepare the pre-metastatic niche, and the gut–brain axis, demonstrating a conserved biological paradigm of distant organ crosstalk. The mechanical and oxidative stress of CPB triggers substantial release of EVs from activated platelets, fragmented erythrocytes, and ischemic vascular tissues [16]. Unlike homeostatic EVs, these surgery-induced vesicles appear to be enriched with pro-coagulant and pro-inflammatory components [17].

**Figure 1:**
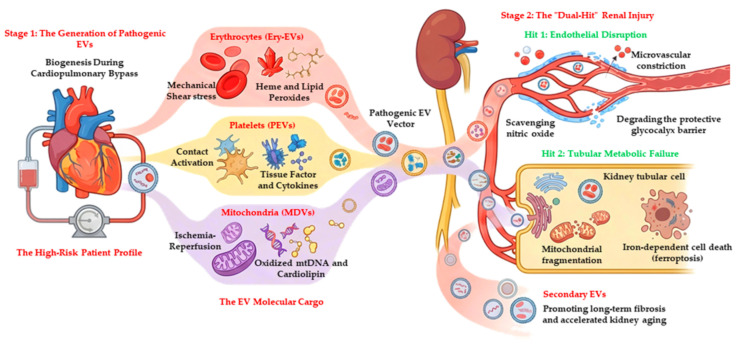
The “Dual-Hit” Pathogenic Mechanism of EV-Mediated Cardiac Surgery-Associated Acute Kidney Injury. The pathophysiology is divided into three conceptual stages to improve clarity. Stage 1 (Generation): The non-physiological forces of cardiopulmonary bypass, including mechanical shear stress, contact activation, and ischemia–reperfusion, trigger the massive release of extracellular vesicles (EVs). Stage 2 (Forms and Markers): Distinct populations emerge, including erythrocyte-derived EVs (Ery-EVs) carrying heme and lipid peroxides, platelet-derived EVs (PEVs) carrying tissue factor, and mitochondrial-derived vesicles (MDVs) transporting oxidized mtDNA. These vesicles function as stable vectors for toxic cargo. Stage 3 (Effects on target cells): This stage outlines the “Dual-Hit” in the kidney. Hit 1: EVs target the renal microvasculature, scavenging nitric oxide and degrading the endothelial glycocalyx, leading to vasoconstriction and barrier loss. Hit 2: Extravasated EVs are internalized by tubular epithelial cells, where they induce mitochondrial fragmentation and iron-dependent cell death (ferroptosis). The cycle culminates in the release of secondary EVs that promote accelerated kidney aging and fibrosis. mtDNA: mitochondrial DNA (Created in BioRender. Vaschetto, R. (2026) https://BioRender.com/1nsjnf9, accessed on 17 April 2026).

Based on this emerging evidence, here we propose a mechanistic model, illustrated in Figure 1, in which pathogenic EVs would contribute to CSA-AKI via a coordinated ‘dual-hit’ mechanism. The first hit targets the vascular interface. The renal endothelium acts as a primary filter for circulating EVs [18]. We hypothesize that heme-rich EVs are internalized by endothelial cells, where they may scavenge NO and uncouple endothelial NO Synthase (eNOS) [19]. Furthermore, EVs carrying transforming growth factor-beta (TGF-β) may initiate endothelial-to-mesenchymal transition (EndMT), a process in which endothelial cells lose their barrier function and acquire a pro-fibrotic phenotype, potentially contributing to microvascular rarefaction [20]. The second hit is proposed to occur within the renal tubular epithelium. As the endothelial barrier is compromised, EVs may penetrate the renal interstitium and be taken up by proximal tubular cells [21]. Here, the EV cargo could exert metabolic toxicity. We propose that EVs transporting oxidized mtDNA may trigger the cyclic guanosin monophosphate–adenosin monophosphate synthase (cGAS) and stimulator of interferon genes (STING) pathway and disrupt mitochondrial dynamics in recipient tubular cells [22]. This would lead to bioenergetic failure, impairing the adenosine triphosphate (ATP)-dependent solute transport that is the kidney’s primary function [23]. Moreover, this stress may suppress cytoprotective factors such as Sirtuin-1 (SIRT1) and Klotho, potentially shifting the kidney into a state of accelerated senescence [24].

While the role of EVs is well-documented in cancer and immunology, their specific function as transducers of surgical stress in CSA-AKI remains underexplored [25]. Much of the mechanistic evidence discussed in this review is derived from experimental models of renal ischemia–reperfusion injury and other critical illnesses, with direct validation in human CSA-AKI representing an important translational gap. This narrative review aims to bridge current knowledge by synthesizing the mechanisms of surgery-induced EV biogenesis, examining the molecular pathogenesis of the proposed “dual-hit” on endothelial and renal tubular cells, and emphasizing the potential role of mitochondrial signaling. Finally, we will discuss future perspectives on targeting the “EV vector” through emerging strategies, including therapeutic apheresis and mitochondria-targeted pharmacotherapy.

## 2. EV Biogenesis and Mechanisms of Release in Cardiopulmonary Bypass

The generation of EVs in CSA-AKI is driven by a dual insult: acute mechanical trauma from the bypass pump combined with sustained biological stress, specifically ischemia–reperfusion injury and systemic inflammation. The initiation of CPB subjects the blood to a constellation of non-physiological forces. These physical and metabolic triggers activate specific cellular signaling pathways within circulating blood cells and the vascular wall, resulting in the rapid shedding of EVs. Clinical evidence indicates a significant increase in circulating EV concentrations during and following CPB. A study by Berckmans et al. demonstrate that this increase mirrors the intensity of hemolysis and inflammatory responses [26], prompting the hypothesis that EV profiles could potentially serve as predictive indicators for the development of postoperative complications like CSA-AKI.

### 2.1. Shear Stress-Induced Hemolysis and Erythrocyte Vesiculation

The mechanical propulsion of blood via roller pumps and centrifugal heads generates zones of high shear stress that are particularly intense at the pump inflow and within the narrow cannulae used for arterial return. This physical trauma contributes directly to overt hemolysis. Concurrently, sub-lethal shear forces induce the generation of erythrocyte-derived EVs (Ery-EVs). It should be noted that while erythrocyte vesiculation under shear stress is well characterized in stored red blood cell models [27], a study directly quantifying the contribution of roller pumps to Ery-EV generation in cardiac surgery patients has not yet been performed.

When erythrocytes are subjected to shear forces exceeding physiological thresholds, their cytoskeleton undergoes deformation that uncouples the lipid bilayer from the spectrin network [28]. This cytoskeletal destabilization triggers calcium-dependent scrambling of membrane phospholipids, resulting in phosphatidylserine externalization on the outer leaflet and subsequent budding of microvesicles from the cell surface [29]. Experimental models and clinical observations indicate that the cardiotomy suction system, which aspirates blood from the surgical field mixed with air and tissue debris, acts as a particularly potent generator of vesicles. The air–blood interface creates surface tension forces that can strip fragments from the erythrocyte membrane [30]. This process generates a population of Ery-EVs that are distinctively enriched in hemoglobin and free heme [31]. Importantly, these vesicles persist in circulation long after the bypass run has ended, serving as a circulating reservoir of pro-oxidant iron that may contribute to delayed organ injury [32].

### 2.2. Contact System Activation and the Platelet Response

Simultaneously, the vast surface area of the extracorporeal circuit triggers the contact activation system. Despite systemic heparinization, the adsorption of plasma proteins, such as fibrinogen and von Willebrand factor, onto artificial surfaces facilitates the adhesion and activation of platelets [33]. Activated platelets undergo profound morphological changes, including actin polymerization and membrane remodeling, which promote the shedding of platelet-derived EVs (PEVs) from their plasma membrane [34].

PEVs represent the most abundant population of circulating vesicles during CPB, often accounting for the majority of the total EV pool in flow cytometric analyses [35]. Unlike the parent platelet, which may become dysfunctional or sequestered during surgery, PEVs remain highly active pro-coagulant effectors. Their membranes are enriched with Tissue Factor and P-selectin, enabling them to bind to leukocytes and endothelial cells and thereby amplify inflammatory signaling throughout the systemic microvasculature [36]. This biogenic pathway is further exacerbated by the re-transfusion of pericardial shed blood, which contains high concentrations of activated PEVs and inflammatory mediators generated in the operative field [37]. Elevated PEV counts correlate with increased postoperative thrombotic and inflammatory complications, although their specific contribution to CSA-AKI remains an active area of investigation [38].

### 2.3. Ischemia–Reperfusion and Tissue-Specific Release

While mechanical forces dominate the maintenance phase of CPB, the cessation of bypass and removal of the aortic cross-clamp introduce a metabolic trigger for EV release: ischemia–reperfusion injury. Reperfusion of the ischemic myocardium results in a rapid burst of reactive oxygen species (ROS) and calcium overload within cardiomyocytes and coronary endothelial cells [39]. This oxidative stress triggers the opening of the mitochondrial permeability transition pore (mPTP) and promotes the release of mitochondrial-derived vesicles (MDVs) directly into the coronary sinus and systemic circulation [40].

Unlike plasma membrane-derived microvesicles, these vesicles are proposed to contain components of the mitochondrial matrix and inner membrane, including oxidized mitochondrial DNA (ox-mtDNA), Cytochrome c, and cardiolipin [41]. While direct isolation and characterization of MDVs from cardiac surgery patients is technically challenging and remain limited, increased circulating levels of cell-free mtDNA and mitochondrial proteins have been detected in the plasma of patients undergoing cardiac surgery, supporting the concept of mitochondrial release during reperfusion [42].

Furthermore, the systemic inflammatory response leads to activation of the peripheral endothelium, resulting in the release of endothelial-derived EVs (Endo-EVs) expressing adhesion molecules such as Intercellular Adhesion Molecule-1 (ICAM-1), Vascular Cell Adhesion Molecule-1 (VCAM-1), and E-selectin [43]. These tissue-specific vesicles may serve as indicators of organ stress, carrying cargo that reflects the metabolic state of the ischemic heart and vasculature [44]. Thus, the patient emerging from CPB possesses a circulating plasma environment that is substantially altered, characterized by a heterogeneous mixture of EVs derived from mechanical damage, contact activation, and metabolic stress [16]. The relative contribution of each EV subtype to renal injury likely varies depending on surgical complexity, bypass duration, and individual patient factors [45].

## 3. Molecular Composition of Surgery-Induced EVs

The pathogenicity of surgery-induced EVs is proposed to be determined by the specific bioactivity of their intra-vesicular and surface-bound cargo. Unlike EVs released under physiological homeostasis, which mediate trophic signaling, vesicles generated during CPB appear to carry a molecular signature of cellular damage. This cargo represents a concentrated profile of the systemic stress response, creating a stable vehicle that may protect labile toxic moieties from plasma clearance mechanisms. Understanding the precise molecular inventory of these vesicles is essential for delineating how they might bypass host defenses to injure the kidney. While comprehensive proteomic and transcriptomic characterization of EVs specifically from CSA-AKI patients remains limited, emerging data from cardiac surgery cohorts and experimental models have begun to define the pathogenic cargo.

### 3.1. Pro-Oxidant Cargo

A prominent characteristic of CPB-induced vesicular profiles is the abundance of Ery-EVs [46]. These vesicles function as protected reservoirs of redox-active iron. Under normal conditions, cell-free hemoglobin released during hemolysis is rapidly bound by the scavenger protein haptoglobin, which targets it for clearance via the CD163 receptor on macrophages [47]. However, studies in hemolytic disorders and transfusion medicine indicate that hemoglobin encapsulated within Ery-EVs is inaccessible to haptoglobin. The lipid bilayer shields the hemoglobin from detoxification, allowing it to circulate for prolonged periods while retaining its oxidative potential [48].

Within the confined lumen of the vesicle, the high concentration of hemoglobin is prone to auto-oxidation, transitioning from ferrous (Fe^2+^) oxyhemoglobin to ferric (Fe^3+^) methemoglobin, which is a process that can lead to the release of free heme and labile iron into the vesicle interior [49]. Furthermore, the membrane of Ery-EVs itself may become a vector of oxidative stress. Due to the high partial pressure of oxygen utilized in the CPB circuit, the polyunsaturated fatty acids of the vesicular membrane can undergo peroxidation, leading to the accumulation of toxic lipid aldehydes such as 4-hydroxynonenal (4-HNE) and malondialdehyde (MDA) on the EV surface [50]. Experimental data suggest that these vesicles may circulate as mobile platforms of lipid peroxidation, capable of transferring reactive lipid species to the plasma membranes of recipient renal cells upon contact. This mechanism could deliver a concentrated payload of catalytic iron and lipid peroxides past the antioxidant defenses of the blood, potentially priming the renal vasculature for injury [51]. However, the relationship between circulating Ery-EV levels, their hemoglobin content, and clinical AKI incidence requires further validation in large prospective cohorts.

### 3.2. Mitochondrial-Derived Damage-Associated Molecular Patterns (mtDAMPs)

In addition to hematologic components, the metabolic stress of ischemia–reperfusion is proposed to release a distinct population of vesicles carrying mitochondrial components. These MDVs may serve as shuttles for DAMPs that possess high immunogenic potency due to their bacterial ancestry. The most critical cargo in this category is ox-mtDNA [52]. Unlike nuclear DNA, mtDNA is rich in unmethylated CpG motifs and lacks protective histones. While this specific oxidative modification of mtDNA has been widely characterized in general ischemia–reperfusion models, we hypothesize that a similar phenomenon occurs during the reperfusion phase of cardiac surgery. Extrapolating from these models, when packaged into EVs, this oxidized genetic material is protected from plasma nucleases such as DNAse I, which would otherwise degrade naked DNA in circulation [53].

Proteomic profiling of plasma EVs from patients undergoing cardiac surgery has identified the presence of specific electron transport chain subunits embedded within the vesicular membrane, including Cytochrome c oxidase and ATP synthase components [54]. Additionally, these vesicles have been found to be enriched with Cardiolipin, a unique dimeric phospholipid normally restricted to the inner mitochondrial membrane that acts as a direct activator of the innate immune system when externalized on the surface of EVs [55]. By encapsulating ox-mtDNA and exposing Cardiolipin, these EVs may function as circulating danger signals. This molecular arrangement could allow them to trigger sterile inflammation in remote tissues without the need for infection, potentially linking the ischemic heart directly to renal tubular inflammation [56]. Nevertheless, direct demonstration that cardiac surgery-derived MDVs are internalized by renal tubular cells and activate inflammatory pathways in vivo remains an important research priority.

### 3.3. Pro-Inflammatory and Epigenetic Cargo

The potential toxic effects of these vesicles may be further amplified by a complex array of signaling molecules that could reprogram recipient cell gene expression. Surgery-induced EVs are enriched with pro-inflammatory cytokines, specifically Interleukin-6 (IL-6), Tumor Necrosis Factor-alpha (TNF-α), and Interleukin-1beta (IL-1β) [57].

Proteomic analyses reveal that these cytokines are often membrane-bound or encapsulated within the vesicle rather than soluble, potentially conferring resistance to proteolytic degradation and increasing their local concentration at the EV–cell interface compared to plasma levels [58]. This may allow EVs to deliver a sustained inflammatory signal that persists longer than the transient spike of soluble cytokines observed immediately after surgery.

At the epigenetic level, specific regulatory microRNAs (miRNAs) have been identified within the EVs of patients developing CSA-AKI, though whether these miRNAs are causal drivers or biomarkers of injury remains to be fully elucidated [59]. These short non-coding RNAs dictate post-transcriptional gene silencing and have been implicated in injury pathways. The EV environment protects these miRNAs from RNase activity via association with Argonaute proteins [60]. miR-21 is upregulated in EVs following renal ischemia and has been detected in cardiac surgery patients. This miRNA targets genes involved in metabolic regulation and fibrosis and has been shown in experimental models to suppress protective pathways such as peroxisome proliferator-activated receptor alpha (PPAR-alpha) [61]. Similarly, miR-155 and miR-34a are enriched in surgery-induced EVs, and in vitro studies indicate they target anti-aging and antioxidant genes, including SIRT1 and Klotho [62]. Furthermore, cardiac-specific miRNAs, such as miR-1 and miR-133a, are released into circulation during myocardial reperfusion. Their detection in urine suggests filtration by the kidney, where they may exert off-target effects on tubular epithelial cells [63]. While correlative studies link specific EV-miRNA profiles to CSA-AKI outcomes, functional validation through gain- or loss-of-function experiments in relevant models is needed to establish causality [64]. Oxidative iron, mtDAMPs, and regulatory RNAs as cargoes of EVs elucidate the potential role of EVs as mediators of perioperative renal pathology [65,66].

However, it is essential to recognize that not all surgery-induced EVs carry pathogenic cargo; protective EVs derived from mesenchymal stromal cells and healthy endothelium are also released and may contribute to tissue repair. The net effect on the kidney likely depends on the balance between pathogenic and reparative vesicle populations, which may vary with surgical complexity, patient comorbidities, and genetic factors [67].

A comprehensive summary of the cellular sources, specific molecular cargo, and pathogenic mechanisms of the cardiac surgery-induced EVs is provided in Table 1.

## 4. EV-Mediated Endothelial Injury Pathophysiology: The First Hit

The renal endothelium serves as the primary barrier between the systemic circulation and the vulnerable tubulointerstitial compartment. Receiving approximately 20% of the cardiac output, the renal microvasculature is continuously exposed to circulating factors, including the EVs generated during CPB. Based on experimental and clinical evidence from related contexts, we propose a model in which pathogenic EVs may contribute to the “first hit” of CSA-AKI by disrupting endothelial homeostasis through three distinct but synergistic mechanisms: NO scavenging, phenotypic transition, and barrier disruption. While direct demonstration of these pathways in human CSA-AKI renal tissue remains limited, the mechanisms are supported by studies in related vascular beds, experimental AKI models, and observational data from cardiac surgery cohorts.

### 4.1. NO Scavenging and Vasomotor Dysregulation

One proposed immediate functional consequence of circulating Ery-EVs is the disruption of vasomotor tone. As detailed in Section 3.1, these vesicles are laden with high concentrations of hemoglobin and heme. In sickle cell disease and hemolytic disorders, upon accumulation in the microcirculation, Ery-EVs are internalized by endothelial cells via CD36 scavenger receptors or extravasate into the subendothelial space [68]. In either location, the encapsulated hemoglobin could act as a potent sink for NO. The dioxygenation reaction between NO and oxyhemoglobin occurs at a constant rate near the diffusion limit (k ≈ 10^7^ M^−1^ s^−1^), which is orders of magnitude faster than the physiological interaction between NO and its downstream target, soluble guanylate cyclase [69].

This rapid reaction can deplete the bioavailable NO pool by converting it into biologically inert nitrate and methemoglobin. The consequent loss of NO-mediated signaling may lead to unopposed vasoconstriction. This effect could be particularly damaging in the renal medulla, where oxygen tension is physiologically low [70]. Furthermore, EV-driven micro-thrombosis and localized coagulation cascades act as major confounding factors. This helps explain the ‘hypertension gap,’ where systemic blood pressure appears normal, but renal microvascular perfusion is critically blocked due to capillary congestion and unopposed constriction. Additionally, the heme cargo delivered by EVs can promote the uncoupling of eNOS. By oxidizing the essential cofactor tetrahydrobiopterin to dihydrobiopterin, EV-derived heme may force the enzyme to switch from producing NO to generating superoxide [71]. This could create a feed-forward loop of oxidative stress and vasoconstriction that persists after the cessation of CPB. However, direct measurement of renal microvascular NO bioavailability and eNOS coupling status in cardiac surgery patients has not yet been performed, representing an important area for future investigation.

### 4.2. Endothelial-to-Mesenchymal Transition (EndMT)

While NO scavenging may drive acute dysfunction, evidence from CKD and fibrosis models suggests that EVs may also initiate a maladaptive process known as EndMT. This phenotypic plasticity allows endothelial cells to de-differentiate, losing their apical–basal polarity and specific markers, such as CD31 and VE-Cadherin, while acquiring a motile, fibroblast-like phenotype characterized by the expression of alpha-smooth muscle actin (α-SMA) and vimentin [72].

Surgery-induced EVs induce EndMT [73]. They transport Transforming Growth Factor β (TGF-β) on their surface, potentially delivering it directly to endothelial receptors, TGFβR1 and TGFβR2. This binding event can trigger the phosphorylation of Smad2 and Smad3. Once phosphorylated, the Smad complex translocates into the nucleus and interacts with transcription factors, such as Snail, Slug, and Twist, which repress the expression of endothelial junction proteins [73]. Simultaneously, in vitro studies have shown that the EV cargo includes specific microRNAs such as miR-21 and let-7, which can silence the expression of key intracellular inhibitors of fibroblast pathways, such as phosphatase and tensin homolog (PTEN) and sprouty RTK signaling antagonist 1 (Spry1) [74].

If this process occurs in the post-cardiac surgery kidney, the biological consequences could be significant. Transition-state cells may migrate into the interstitium and contribute to fibrosis. More critically, the loss of functional endothelial cells could lead to microvascular rarefaction, a permanent reduction in capillary density [75]. This structural loss may compromise the ability of the kidney to re-oxygenate during recovery and could predispose patients to the transition from acute kidney injury to CKD. Direct histological evidence of EndMT in human renal biopsies from CSA-AKI patients is currently lacking, though microvascular rarefaction has been documented in chronic settings following cardiac surgery [76].

### 4.3. Glycocalyx Shedding and Barrier Permeability

The third component of endothelial injury is the physical degradation of the permeability barrier. The endothelial glycocalyx is a gel-like layer of proteoglycans and glycoproteins that lines the luminal surface, regulating permeability and preventing leukocyte adhesion [77]. Clinical studies have documented that EVs released during CPB may be enriched with enzymes, such as heparanase and hyaluronidase, or may induce endothelial expression of matrix metalloproteinases via the Nuclear Factor kappa-light-chain-enhancer of activated B cells (NF-κB) signaling pathway [78].

The activity of these EV-associated enzymes can result in shedding of the glycocalyx. This phenomenon is supported by elevated serum levels of syndecan-1, a core glycocalyx component, in cardiac surgery patients [79]. Furthermore, syndecan-1 (SDC1) levels have been shown to correlate with the severity of postoperative complications, including AKI, in some cohorts [80]. The denudation of this protective layer exposes adhesion molecules such as ICAM-1 and VCAM-1, facilitating the firm arrest and transmigration of neutrophils and monocytes into the renal parenchyma.

Presumably, the downregulation of zonula occludens-1 (ZO-1) by EV-derived miR-155 would lead to the loss of the glycocalyx and the disruption of inter-endothelial tight junctions, which ultimately would cause a drastic increase in vascular permeability [81].

This could create a leaky barrier that allows protein-rich plasma and toxic circulating factors, including the EVs themselves, to extravasate into the interstitial space.

## 5. Tubular Epithelial Cell Injury and Metabolic Failure: The Second Hit

Following the disruption of the endothelial barrier, pathogenic EVs may traverse the compromised peritubular capillary wall and enter the renal interstitium. Here, they could be internalized by proximal tubular epithelial cells (PTECs). While PTECs possess high endocytic capacity via the megalin/cubilin receptor complex to reclaim filtered proteins, the same machinery may render them vulnerable to the uptake of pathogenic vesicles accumulating in the interstitial space [82]. PTECs are the metabolic workhorse of the nephron, responsible for the reabsorption of approximately 65% of the filtered solute load. This immense transport activity is entirely dependent on a continuous supply of ATP generated by oxidative phosphorylation [83]. Consequently, PTECs are among the most mitochondria-rich cells in the body and are exquisitely vulnerable to metabolic insults. We propose that the cargo delivered by surgery-induced EVs may target mitochondrial machinery, potentially initiating a cascade of bioenergetic failure, oxidative cell death, and premature senescence that constitutes the “second hit” of CSA-AKI. While these mechanisms are well established in experimental models of renal ischemia–reperfusion injury, their direct demonstration in human cardiac surgery patients remains an important area of ongoing investigation.

### 5.1. Mitochondrial Dynamics and Bioenergetics

Mitochondria in the kidney exist as a dynamic network that constantly undergoes fission and fusion. This balance is critical for maintaining a healthy mitochondrial population. Fusion allows for the mixing of mitochondrial contents and the mitigation of damaged components, whereas fission segregates irreparably damaged mitochondria for clearance via mitophagy [84]. In experimental models of AKI, this balance is acutely disrupted and can shift towards pathological fission, a process that may be exacerbated by EV cargo [85].

The proposed mechanism by which EVs may trigger this shift is centered on the cytosolic activation of dynamin-related protein 1 (Drp1). In renal ischemia–reperfusion models, ox-mtDNA is recognized by cytosolic sensors, such as the cGAS-STING pathway [86]. Activation of STING has been shown to trigger innate immune signaling and promote the dephosphorylation of Drp1 at its inhibitory serine residue (Ser637). This post-translational modification activates Drp1, which then translocates from the cytosol to the outer mitochondrial membrane, where it interacts with receptors such as mitochondrial fission 1 protein (Fis1) and mitochondrial fission factor (Mff) to constrict the organelle, severing the network into fragmented and dysfunctional units [87]. Whether EV-delivered mtDNA specifically drives this pathway in cardiac surgery-associated AKI remains to be directly demonstrated, though the presence of circulating mtDNA has been documented in CPB patients [88].

Mitochondrial fragmentation can have significant consequences for cellular bioenergetics. Fragmented mitochondria often exhibit reduced capacity for oxidative phosphorylation because the electron transport chain complexes function most efficiently within a fused and continuous network that allows for rapid distribution of membrane potential [89]. The resulting decline in ATP synthesis could directly impair the function of Na^+^/K^+^-ATPase and other ATP-dependent transporters on the PTEC basolateral membrane. The loss of this pump activity would disrupt the osmotic gradients required for solute and water reabsorption, potentially leading to clinical manifestations of tubular dysfunction, such as increased fractional excretion of sodium [90]. Furthermore, fragmented mitochondria may become increasingly permeable and release Cytochrome c into the cytosol, potentially amplifying apoptotic signaling in a feed-forward manner [91].

### 5.2. Heme-Induced Ferroptosis and Redox Stress

While mitochondrial fragmentation may contribute to bioenergetic collapse, the pro-oxidant cargo of Ery-EVs could also drive a distinct form of regulated cell death known as ferroptosis. Ferroptosis is an iron-dependent, non-apoptotic cell death pathway characterized by the uncontrolled peroxidation of polyunsaturated fatty acids (PUFAs) in cellular membranes [92]. Due to high lipid content and metabolic activity, PTECs may be particularly susceptible to this mode of death.

The initiation of ferroptosis requires two key elements: labile iron and the failure of antioxidant defenses. Ery-EVs provide a potent source of the former. Upon internalization by PTECs, the hemoglobin and free heme within the EV lumen could be released into the cytosol. Heme is catabolized by the enzyme Heme Oxygenase-1 (HO-1) to yield Fe^2+^. While HO-1 is initially cytoprotective, experimental data indicate that overwhelming induction by high heme loads can paradoxically lead to accumulation of intracellular labile iron that exceeds the storage capacity of ferritin [93]. This excess labile iron can catalyze the Fenton reaction, generating hydroxyl radicals that attack PUFA membranes and initiate a chain reaction of lipid peroxidation.

Under normal conditions, the phospholipid hydroperoxidase, glutathione peroxidase 4 (GPX4), serves as the central guardian against ferroptosis. GPX4 utilizes reduced glutathione (GSH) as a cofactor to convert toxic lipid hydroperoxides into non-toxic lipid alcohols. However, the oxidative stress potentially delivered by EVs could deplete the GSH pool. GSH synthesis is dependent on the import of cystine via the System Xc^−^ antiporter. In vitro studies suggest that inflammatory cytokines and oxidative stress can suppress the expression of this transporter, limiting cystine uptake and depleting GSH [94]. Furthermore, experimental evidence indicates that reactive lipid aldehydes such as 4-HNE can directly conjugate to and inactivate GPX4 [95]. If these defenses are neutralized, the PTECs may succumb to uncontrolled membrane damage, potentially leading to cell membrane rupture and release of intracellular contents that further propagate inflammation. While ferroptosis has been demonstrated in experimental models of AKI, direct evidence of ferroptotic cell death in renal biopsies from cardiac surgery patients is currently lacking [96].

### 5.3. Accelerated Senescence and Suppression of Anti-Aging Factors

Beyond acute cell death, a subset of PTECs subjected to sub-lethal EV-mediated injury may enter a state of cellular senescence. Senescent cells are characterized by permanent cell cycle arrest, rendering them incapable of proliferation and tissue regeneration [97]. While initially this could represent a protective mechanism to prevent the replication of damaged cells, the accumulation of senescent cells can promote fibrosis and chronic inflammation of surrounding tissue via the senescence-associated secretory phenotype (SASP) [98].

While direct evidence linking surgery-induced EVs to the suppression of SIRT1 and Klotho in vivo is currently lacking, we extrapolate from related oxidative stress models that EV-mediated delivery of pathogenic miRNAs promotes this senescent phenotype by suppressing the expression of key anti-aging factors. SIRT1 is a Nicotinamide Adenine Dinucleotide (NAD^+^)-dependent deacetylase that promotes cellular resilience by activating Peroxisome proliferator-activated receptor gamma coactivator 1-alpha (PGC-1α), the master regulator of mitochondrial biogenesis [99]. In vitro and animal studies have shown that pathogenic miRNAs, particularly miR-34a and miR-155, can directly target the 3′-untranslated region of SIRT1 mRNA, leading to its degradation and translational repression [100]. The resulting loss of SIRT1 could impair the ability of the PTECs to regenerate their mitochondrial network, potentially locking the cell into a senescent state.

Similarly, Klotho is a transmembrane protein predominantly expressed in the distal tubule that functions as a powerful anti-aging and anti-fibrotic factor. Soluble Klotho exerts pleiotropic renoprotective effects, including the suppression of TGF-β signaling and the inhibition of oxidative stress [101]. Multiple stressors associated with AKI have been shown to suppress Klotho gene transcription in experimental models [102]. Furthermore, in vitro studies suggest that miR-21 can target and silence Klotho mRNA [103]. The loss of Klotho could remove a critical brake on the fibrotic pathways activated by the endothelial injury described previously, potentially creating a convergent state where both the vascular and tubular compartments are primed for chronic fibrosis [104]. While correlative clinical data link decreased Klotho levels to worse outcomes following cardiac surgery, whether EV-delivered miRNAs are the direct mechanism remains to be established [105]. The proposed EV-mediated suppression of SIRT1 and Klotho may represent a plausible mechanism by which an acute surgical insult could be transduced into a long-term trajectory toward CKD, though prospective interventional studies targeting these pathways are needed to establish causality [106].

## 6. Intercellular Crosstalk and Injury Propagation: The Amplification Loop

The previous sections delineated the distinct injuries sustained by the endothelium and the renal tubular epithelium. However, the renal microenvironment is a tightly integrated ecosystem where these compartments are separated only by a sub-endothelial basement membrane and a narrow interstitial space. The pathophysiology of CSA-AKI is, therefore, likely not a static series of independent hits but a dynamic, evolving process potentially driven by paracrine communication. While direct evidence in cardiac surgery patients is limited, injured cells within the kidney in other disease contexts do not merely succumb to toxicity; they become active sources of secondary pathogenic signals. Drawing on insights from CKD and experimental AKI models, we propose a conceptual framework in which a “secondary wave” of tissue-derived EVs may propagate damage from the vascular compartment to the tubule and recruit the innate immune system into a self-amplifying loop of injury. Testing this framework in the specific context of CSA-AKI may represent an important future research direction.

### 6.1. Endothelial-to-Tubular Signaling: The Loss of Trophic Support

Experimental evidence suggests that endothelial cells subjected to oxidative and shear stress can undergo a profound secretory phenotype shift. In healthy conditions, the peritubular capillary endothelium provides essential trophic support to adjacent proximal tubules via the secretion of vascular endothelial growth factor (VEGF) and NO [107]. However, studies in models of CKD and vascular injury indicate that under stress, including potentially EV-mediated injury and EndMT, endothelial cells may release a distinct population of vesicles that could subvert tubular survival pathways [108].

Proteomic and transcriptomic analyses of endo-EVs in other contexts have revealed enrichment with pro-apoptotic signals and miRNAs that target tubular integrity. Injured endothelial cells release EVs containing elevated levels of the miR-200 family and components of the TGF-β signaling pathway [109]. Upon uptake by adjacent PTECs, these vesicles have been shown to suppress the expression of anti-apoptotic proteins, such as B-cell lymphoma 2 (Bcl-2) and survivin, potentially sensitizing renal tubular cells to cell death even in areas where perfusion may be partially preserved [110]. Furthermore, studies in cancer and fibrosis models suggest that EVs can transport phosphorylated mothers against decapillogic homolog (Smad) proteins directly to recipient cells. This horizontal transfer of signaling complexes may bypass the need for ligand–receptor binding and could directly activate pro-fibrotic gene programs in the tubule [111].

It has been proposed that this crosstalk also involves the interruption of protective signals. The loss of endothelial continuity (rarefaction) may disrupt the delivery of angiocrine factors that normally maintain tubular quiescence [112]. The replacement of trophic signals with pro-fibrotic EV cargo could drive tubular cells into a dedifferentiated, secretory state. This paracrine mechanism is consistent with the clinical observation that tubular injury is often most severe in topographic regions adjacent to rarefied peritubular capillaries, suggesting that the endothelium may act as a transducer that converts a systemic blood-borne signal into localized tissue-specific injury [113]. However, direct demonstration of endothelial-to-tubular EV transfer and its functional consequences in human or experimental CSA-AKI is currently lacking.

### 6.2. Tubular-to-Immune Signaling: Macrophage Polarization

Conversely, experimental data suggest that injured PTECs may engage in retrograde signaling to the interstitium and the immune system. In models of AKI, renal tubular cells undergoing cell death have been shown to release EVs carrying tissue-specific DAMPs, including high mobility group box 1 and heat shock proteins [114]. Studies indicate that these tubular-derived EVs can be internalized by resident renal macrophages and dendritic cells surveilling the interstitium [115].

The internalization of DAMP-laden vesicles triggers functional polarization of macrophages. While resident macrophages typically exhibit an anti-inflammatory M2 phenotype involved in tissue repair, experimental evidence suggests that uptake of injured tubular EVs may induce a metabolic shift towards the pro-inflammatory M1 phenotype [116]. Mechanistically, in cell culture studies, this has been attributed to vesicular transfer of specific miRNAs, such as miR-19b-3p and miR-23a, which can target and suppress macrophage signaling inhibitors, such as PTEN and A20, thereby potentially unleashing NF-κB activation [117].

In experimental AKI models, activated M1 macrophages secrete high levels of TNF-α, IL-1β, and ROS, which can cause collateral damage to neighboring healthy tubules and further degrade the peritubular capillary endothelium [118]. This could create a bidirectional feedback loop: endothelial injury may promote tubular death, dying tubules may activate macrophages, and activated macrophages could damage the endothelium [119]. If operative in CSA-AKI, this “vicious cycle” would sustain the inflammatory milieu within the kidney for days or weeks after the initial surgical insult has resolved, preventing resolution of injury. However, whether this specific EV-mediated crosstalk occurs in cardiac surgery patients, and whether it drives clinical outcomes, requires validation in translational studies.

### 6.3. Pericyte Detachment and Maladaptive Repair

The proposed ultimate consequence of persistent crosstalk is the activation of renal stromal cells, specifically pericytes. Pericytes are mural cells that wrap around endothelial cells to stabilize capillary structure. The interplay between inflammatory signals creates a microenvironment rich in platelet-derived growth factor-B and TGF-β signaling, which can trigger pericyte detachment [120].

Experimental studies have shown that once detached from the capillary wall, pericytes can undergo myofibroblast transition, migrating into the interstitial space where they proliferate and deposit collagen types I and III [121]. In models of renal fibrosis, this process appears to be accelerated by developmental signaling pathways. The Sonic Hedgehog (SH) pathway is a potent driver of fibroblast proliferation in these contexts [122]. While direct evidence is limited, it has been proposed that injured tubular cells may release SH ligands within EVs, potentially promoting the transdifferentiation of pericytes into scar-forming myofibroblasts [123].

This model of persistent fibrotic signaling could drive the transition from AKI to CKD. In chronic settings, deposition of extracellular matrix widens the distance between capillaries and tubules, worsening hypoxia and potentially perpetuating the cycle of injury [124]. Furthermore, as discussed in Section 5.3, senescent renal tubular cells locked in cell cycle arrest may secrete profibrotic factors that prevent re-establishment of normal tubular architecture [125]. In this framework, the “EV vector” could be responsible not only for the acute necrotic phase of CSA-AKI but may also orchestrate maladaptive repair processes that lead to permanent renal dysfunction [126]. However, the extent to which these mechanisms operate in the specific temporal and metabolic context of cardiac surgery remains to be elucidated. Prospective studies examining EV content, recipient cell types, and functional outcomes in CSA-AKI patients are critically needed to test this proposed amplification loop.

## 7. Therapeutic Perspectives and Clinical Translation

The mechanistic understanding of EV-mediated pathogenesis outlined in this review suggests multiple potential therapeutic targets. The goal of intervention would be to disrupt the pathological cascade at its earliest stages, ideally before the proposed establishment of a self-sustaining feedback loop. Emerging strategies can be broadly categorized into three approaches: the physical removal of pathogenic EVs and their cargo from the circulation, pharmacological neutralization of toxic effects within target cells, and the use of EVs as diagnostic biomarkers for early risk stratification. While each approach shows theoretical promise, translation to clinical practice faces significant technical, regulatory, and economic challenges.

### 7.1. Extracorporeal Blood Purification

Extracorporeal blood purification offers an approach to remove pathogenic vesicles before they reach the kidney. This strategy aligns with the existing clinical infrastructure of cardiac surgery, as the CPB circuit can be modified to incorporate blood purification technologies. Hemoadsorption devices such as the CytoSorb cartridge have been deployed in cardiac surgery settings with the primary aim of reducing circulating cytokines [127]. These devices utilize porous polymer beads with high surface area to adsorb molecules within a specific size range (5–60 kDa) [128].

These cartridges effectively remove a fraction of smaller EVs and soluble cargo, including cell-free hemoglobin and inflammatory mediators [129]. The integration of such devices into the CPB circuit is technically feasible and has been evaluated in several clinical trials. However, results have been mixed. Some single-center studies report reductions in postoperative IL-6 levels and vasopressor requirements [130]; conversely, large multicenter randomized controlled trials have failed to demonstrate consistent benefits in clinical outcomes, including AKI incidence or mortality [131]. A recent meta-analysis concluded that while CytoSorb may reduce inflammatory markers, evidence for improved patient-centered outcomes remains insufficient [132].

Furthermore, current hemoadsorption technology is not specifically designed for EV removal, and its effects on the EV pool remain poorly characterized. Future iterations could incorporate membrane filters with pore sizes optimized to capture microvesicles (100–1000 nm) while allowing essential plasma proteins to pass through [133]. Additionally, affinity-based columns targeting specific EV surface markers, including CD235a for Ery-EVs or CD41 for PEVs, could trigger a more selective purification strategy, potentially removing pathogenic vesicles while sparing beneficial homeostatic EVs [134].

However, such technologies remain experimental, and their development faces challenges including cost, biocompatibility, and the risk of removing protective EVs alongside pathogenic ones [135]. These extracorporeal approaches, while theoretically attractive, require rigorous validation in adequately powered clinical trials before routine clinical implementation can be recommended.

### 7.2. Mitochondrial and Metabolic Pharmacotherapy

For patients in whom EV exposure has already occurred, pharmacological intervention may target downstream intracellular effects. Given the central role of mitochondrial dysfunction described in Section 5, mitochondria-targeted therapies would represent a rational, though still investigational, approach. In this context, a leading candidate is Elamipretide (SS-31; also known as MTP-131 or STBA-001), a small tetrapeptide that selectively targets and binds to cardiolipin on the inner mitochondrial membrane [136]. By stabilizing cardiolipin-Cytochrome c interactions, SS-31 optimizes electron transport chain function, reduces ROS production, and prevents opening of the mPTP, thereby limiting mtDNA release [137].

Perioperative SS-31 administration confers significant protection of tubular structure and function in rodent models [138]. In humans, a Phase II randomized controlled trial (EMBRACE STEMI) evaluated SS-31 in patients undergoing percutaneous coronary intervention for acute myocardial infarction and reported a reduction in infarct size as measured by cardiac magnetic resonance [139]. However, a subsequent Phase IIb trial in heart failure (PROGRESS-HF) did not reach its primary endpoint [140]. To date, no adequately powered randomized trial has evaluated Elamipretide specifically for the prevention of CSA-AKI, and its application in this context remains investigational [141].

Targeting ferroptosis offers another potential avenue of intervention. Iron chelators, such as Deferoxamine or the more lipophilic Deferiprone, can sequester the labile iron pool generated by heme catabolism, potentially preventing it from catalyzing the Fenton reaction [142]. Iron chelation protects against ischemia–reperfusion-induced tubular ferroptosis in rodent models [143]. Additionally, compounds such as Liproxstatin-1 and Ferrostatin-1 act as potent lipophilic radical-trapping antioxidants, capable of directly terminating lipid peroxidation chain reactions at the membrane independent of GPX4 activity [144]. However, these agents are currently preclinical research tools and are not approved for clinical use in any indication. Their pharmacokinetics, toxicity profiles, and optimal dosing in humans remain undefined [145]. Therefore, while the ferroptosis pathway represents a compelling therapeutic target, significant translational work is required before these inhibitors can be tested in human trials.

### 7.3. EV-Based Diagnostics and Risk Stratification

The current diagnosis of AKI relies on serum creatinine, which is a marker with well-recognized limitations. Creatinine rises only after substantial functional impairment has occurred, typically 24 to 48 h after the insult, thereby precluding early intervention [146]. EVs have been proposed as real-time liquid biopsies of ongoing pathology, offering the potential for earlier detection [147].

In principle, quantification and characterization of circulating EVs using flow cytometry or nanoparticle tracking analysis (NTA) could provide an early warning signal of impending injury. A surge in Ery-EVs or PEVs during or immediately after CPB might identify patients at highest risk before changes in creatinine are detectable [148]. Furthermore, elevated levels of EV-encapsulated ox-mtDNA or specific miRNAs, including miR-21 as EV cargo, could provide mechanistic information and serve as a biomarker signature for the proposed “dual-hit” pathway [149].

However, significant technical and regulatory barriers currently limit clinical implementation. EV isolation and quantification methods lack standardization, with different techniques (ultracentrifugation, size-exclusion chromatography, precipitation kits) yielding inconsistent results [150]. Both flow cytometry and NTA have technical limitations in sensitivity and specificity for small EVs (<200 nm) [151]. Furthermore, no EV-based biomarker assay has been approved by the Food and Drug Administration for clinical use in AKI, and prospective validation studies defining clinically actionable cutoff values are lacking [152]. Large, multicenter cohort studies with harmonized protocols are needed to establish the diagnostic accuracy, clinical utility, and cost-effectiveness of EV-based risk stratification in cardiac surgery patients [153]. Resolving these barriers is a prerequisite before personalized EV-based perioperative strategies can move from concept to practice. Beyond diagnostics, EVs also present a therapeutic opportunity through administration of MSC-EVs, which carry a fundamentally different cargo from the pathogenic vesicles generated during CPB. MSC-EVs are enriched with growth factors, antioxidant proteins, and regulatory miRNAs that promote tubular cell regeneration, reduce oxidative stress, and suppress inflammation across preclinical AKI models [154,155]. Exosome-enriched populations derived from bone marrow MSCs have further shown capacity to stimulate tubular proliferation, inhibit apoptosis, and restore mitochondrial function [156], representing a biologically grounded counterpart to the harmful EV signaling that drives CSA-AKI.

Current and emerging therapeutic strategies targeting this EV-mediated injury cascade are summarized in Table 2.

## 8. Limitations and Research Gaps

While this review synthesizes mechanistic evidence from diverse experimental and clinical studies, several critical limitations must be acknowledged. First, the majority of proposed pathways are extrapolated from CKD, cancer, and sepsis models rather than being directly demonstrated in cardiac surgery patients. This is particularly true for pathways involving EV-mediated crosstalk between endothelial, tubular, and immune cells. Rigorous validation in human CSA-AKI cohorts through serial EV profiling, renal tissue analysis, and correlative clinical outcomes is urgently needed.

Second, the inherent heterogeneity of the EV population presents a major methodological challenge. Current isolation techniques (ultracentrifugation, polymer precipitation) co-isolate diverse vesicle subtypes along with non-vesicular contaminants, making it difficult to attribute pathogenicity to specific EV populations [157]. Standardization of isolation methods according to Minimal Information for Studies of Extracellular Vesicles 2018 (MISEV2018) guidelines and the use of high-resolution techniques, such as immunoaffinity capture and single-vesicle analysis, are essential for reproducible biomarker development [158]. Moreover, most clinical studies remain observational and correlative, demonstrating associations between circulating EVs and AKI but not definitive causality. Proof-of-concept studies in which patient-derived EVs are isolated and infused into preclinical models to reproduce the injury phenotype are needed to establish causality [159].

Third, therapeutic strategies targeting EVs must address the critical issue of selectivity. Homeostatic EVs released from endothelial progenitor cells and mesenchymal stem cells carry reparative cargo essential for tissue recovery [160]. Non-selective removal via hemoadsorption may inadvertently deplete these protective vesicles, potentially impairing renal regeneration. Future interventions must aim for selective capture or neutralization of pathogenic EV subtypes (e.g., heme-rich Ery-EVs, pro-coagulant PEVs) while preserving the beneficial vesicular secretome [161]. To substantiate the proposed ‘two-hit’ hypothesis and resolve the confounding effects of heterogeneous EV populations, future research must pivot to advanced preclinical models. Utilizing 3D human renal organoids or microfluidic ‘kidney-on-a-chip’ systems would allow researchers to isolate specific EV sources (e.g., purely erythrocyte-derived vs. platelet-derived) and observe their distinct pro- or anti-inflammatory effects on endothelial and tubular structures in real time [162].

Finally, significant translational gaps remain. The optimal timepoints for EV sampling, the dose–response relationship between EV burden and injury severity, and the influence of patient-specific factors (genetics, comorbidities, surgical complexity) on EV-mediated pathophysiology are poorly understood. Large, multicenter prospective cohorts with standardized protocols are required to translate EV biology from bench to bedside and to determine whether EV-targeted diagnostics and therapeutics can improve clinical outcomes in cardiac surgery patients [163].

## 9. Conclusions

CSA-AKI remains a significant clinical challenge whose pathophysiology extends beyond hemodynamic instability alone. This review proposes an integrative mechanistic framework in which the CPB circuit triggers the release of pathogenic EVs from activated platelets, damaged erythrocytes, and stressed vascular tissues. These vesicles may function as stable vectors that deliver pro-oxidant hemoglobin derivatives, immunogenic mitochondrial components, and regulatory microRNAs to the renal parenchyma.

We propose that these EVs may contribute to CSA-AKI through a coordinated “dual-hit” mechanism. The first hit targets the renal endothelium, where EV cargo may scavenge NO, promote EndMT, and degrade the glycocalyx barrier. The second hit targets the proximal tubular epithelium, where EV-delivered signals may induce mitochondrial fragmentation, trigger ferroptotic cell death, and suppress cytoprotective factors, such as SIRT1 and Klotho. Injured cells may subsequently release secondary EVs that amplify inflammation and promote the transition from acute injury to chronic fibrosis.

While this framework is supported by experimental evidence and clinical associations, direct validation in human CSA-AKI remains incomplete. Future research priorities would include establishing causality through EV transfer studies, identifying pathogenic EV subtypes using standardized isolation methods, and developing clinically actionable biomarker panels. Therapeutic strategies targeting this pathway may offer promising avenues but require rigorous clinical validation. These strategies include selective extracorporeal EV removal, mitochondria-targeted pharmacotherapy, and EV-based risk stratification.

Addressing these challenges through collaborative translational research may ultimately enable a precision medicine approach to protecting the kidney during cardiac surgery.

## Figures and Tables

**Table 1:** Sources, Cargo, and Pathogenic Mechanisms of Surgery-Induced Extracellular Vesicles.

EV Source and Trigger	Key Molecular Cargo	Pathogenic Mechanism and Renal Outcome	References
Erythrocytes (Ery-EVs) (Trigger: Shear stress, Hemolysis)	Cell-free Hemoglobin; Free Heme; Lipid Peroxides (4-HNE)	Scavenges NO causing vasoconstriction; Heme overload triggers tubular ferroptosis.	[27,31,46,49]
Platelets (PEVs) (Trigger: Contact activation, Heparin)	Tissue Factor; P-Selectin; Inflammatory Cytokines (IL-1β)	Promotes leukocyte recruitment and microthrombosis, leading to microvascular congestion.	[33,35,36]
Mitochondria (MDVs) (Trigger: Ischemia- Reperfusion)	Oxidized mtDNA; Cardiolipin; Cytochrome c	Activates cGAS-STING pathway and Drp1-mediated fission, causing bioenergetic failure.	[40,41,54,55]
Endothelium (Endo-EVs) (Trigger: Oxidative stress, Shear)	TGF-β; Adhesion molecules; Pathogenic miRNAs (miR-21, miR-155)	Induces EndMT and suppresses anti-aging factors (SIRT1/Klotho), driving fibrosis and senescence.	[43,61,62,63]

4-HNE: 4-hydroxynonenal; cGAS-STING: cyclic GMP-AMP synthase–stimulator of interferon genes; Drp1: dynamin-related protein 1; EndMT: Endothelial-to-Mesenchymal Transition; miRNA: microRNA; mtDNA: mitochondrial DNA; NO: Nitric Oxide; SIRT1: Sirtuin-1; TGF-β: Transforming Growth Factor beta.

**Table 2:** Emerging Therapeutic Strategies Targeting the EV-Mediated Injury Cascade.

Therapeutic Approach	Key Agents/Devices	Mechanism of Action	Current Clinical Status	References
Extracorporeal Blood Purification	Hemoadsorption (e.g., CytoSorb) Affinity Columns (Experimental)	Removal of circulating EVs and cytokines via polymer adsorption or specific surface marker capture (e.g., anti-CD235a).	Clinically available (CytoSorb); Mixed efficacy in AKI trials.	[127,129,134]
Mitochondrial Protection	Elamipretide (SS-31)	Binds cardiolipin on inner mitochondrial membrane to stabilize cristae, prevent ROS generation, and inhibit mtDNA release.	Phase 3 trials in Heart Failure; Investigational for AKI protection.	[136,137,138]
Ferroptosis and Redox Inhibition	Deferoxamine (Iron Chelators) Liproxstatin-1 (Antioxidants)	Sequesters catalytic free iron to prevent Fenton reaction; Directly terminates toxic lipid peroxidation chains.	Iron chelators are FDA-approved; Liproxstatins are preclinical tools.	[142,143,144,145]
Epigenetic Modulation	Anti-miRNA Oligonucleotides	Targeted silencing of pathogenic EV-delivered miRNAs (e.g., anti-miR-21, anti-miR-155) to restore protective factors.	Preclinical development; Early phase trials in fibrosis/Alport syndrome.	[59,100]

AKI: Acute Kidney Injury; EV: Extracellular Vesicle; FDA: Food and Drug Administration; miRNA: microRNA; mtDNA: mitochondrial DNA; ROS: Reactive Oxygen Species.

## Data Availability

Data sharing is not applicable to this article as no new data were created or analyzed in this study. All information discussed is sourced from the publications cited in the reference list.
